# An Android Malware Detection Approach to Enhance Node Feature Differences in a Function Call Graph Based on GCNs

**DOI:** 10.3390/s23104729

**Published:** 2023-05-13

**Authors:** Haojie Wu, Nurbol Luktarhan, Gaoqi Tian, Yangyang Song

**Affiliations:** 1 School of Software, Xinjiang University, Urumqi 830091, China; 107552104325@stu.xju.edu.cn (H.W.); t_gaoqi@stu.xju.edu.cn (G.T.); 2 College of Information Science and Engineering, Xinjiang University, Urumqi 830046, China; song_yy@stu.xju.edu.cn

**Keywords:** Android malware detection, function call graph, TF–IDF, self-loop, graph convolutional network

## Abstract

The smartphone has become an indispensable tool in our daily lives, and the Android operating system is widely installed on our smartphones. This makes Android smartphones a prime target for malware. In order to address threats posed by malware, many researchers have proposed different malware detection approaches, including using a function call graph (FCG). Although an FCG can capture the complete call–callee semantic relationship of a function, it will be represented as a huge graph structure. The presence of many nonsensical nodes affects the detection efficiency. At the same time, the characteristics of the graph neural networks (GNNs) make the important node features in the FCG tend toward similar nonsensical node features during the propagation process. In our work, we propose an Android malware detection approach to enhance node feature differences in an FCG. Firstly, we propose an API-based node feature by which we can visually analyze the behavioral properties of different functions in the app and determine whether their behavior is benign or malicious. Then, we extract the FCG and the features of each function from the decompiled APK file. Next, we calculate the API coefficient inspired by the idea of the TF–IDF algorithm and extract the sensitive function called subgraph (S-FCSG) based on API coefficient ranking. Finally, before feeding the S-FCSG and node features into the GCN model, we add the self-loop for each node of the S-FCSG. A 1-D convolutional neural network and fully connected layers are used for further feature extraction and classification, respectively. The experimental result shows that our approach enhances the node feature differences in an FCG, and the detection accuracy is greater than that of models using other features, suggesting that malware detection based on a graph structure and GNNs has a lot of space for future study.

## 1. Introduction

With the rapid development of technologies, the smartphone has become an indispensable tool in our daily lives. The mainstream operating systems of smartphones available on the market are Android and iOS, and the Android operating system dominates the market. As the Android operating system is popular, it is also the preferred malware target. In 2021, Google Play users worldwide downloaded 111.3 billion mobile apps, and this was up from 76 billion apps in 2018 [1]. Many malicious apps are hidden in Google Play Store, stealing and modifying the information of users without their authorization. Some even go so far as to hijack users’ smartphones and force them to conduct financial transactions.

Many security technology companies provide Android antivirus products to help people defend themselves against malware. In January 2019, AV-Comparatives downloaded 250 anti-malware security apps from Google Play Store that were created by various developers to test the effectiveness of their anti-malware programs [2]. The test results showed that 80 anti-malware security apps were able to detect more than 30% of malicious applications with zero false alarms, 138 of the remaining 170 apps detected less than 30% of Android malware samples, and 32 apps were deleted from Google Play Store. In reality, the detection efficiency of many Android antivirus products is low when offline, and the greatest detection efficiency can only be achieved when they connect to the online dataset.

To address threats posed by malware, researchers have studied a large number of features targeting Android malware using feature engineering, which can be broadly classified into three main categories: static features, dynamic features, and hybrid features. Static analysis, dynamic analysis, and hybrid analysis are the analysis methods used to obtain these three types of features [3]. The static analysis method analyses the application and its associated objects without executing the application [4]. The dynamic analysis method analyses features while the application is in use (on a real device or in a virtual environment) [4]. The hybrid analysis method is an integrated approach that combines static and dynamic features in different forms and includes a much more comprehensive range of feature dimensions.

In recent years, neural networks have been adapted to leverage the structure and properties of graphs [5], but the traditional neural network models, such as CNNs [6] and LSTMs [7], cannot directly use the graph structure as the input. The emergence of graph neural networks (GNNs) is an excellent solution to this problem. As one of the most commonly used models of GNNs, graph convolutional neural networks (GCNs) update the features of their nodes in the next layer by aggregating the features of neighboring nodes during forward propagation, which can be seen as a special form of low-pass filtering [8,9]. The features of GCNs based on low-pass filtering retain the commonality of node features in the graph and inevitably ignore the differences, making the learned node features similar, and the original features of the important nodes are lost [10]. The function call graph with node features is aggregated with the features of neighbor nodes after the forward propagation of the GCN model, as the low-pass filtering characteristic causes the node features in the function call graph to be similar, resulting in possibly losing the important original features of the nodes. This is not beneficial for model identification and detection. Is there an approach to reduce the interference of this characteristic and enhance node feature differences, thus improving the detection of the model?

In this paper, we extract the function call graph (FCG) from a decompiled APK, which could automatically capture the semantic relationships between different functions and extract three types of features from each function as node features based on the newest API permissions and graph structure. Then, we calculate the API coefficient that can represent the importance of each API and extract the sensitive function called subgraph (S-FCSG) based on the API coefficient ranking. The S-FCSG reduces the number of nonsensical nodes, maximizes the retention of node features, and avoids the convergence of important node features into meaningless node features during the forward propagation of the GCN model. Finally, we add the self-loop to each node of the S-FCSG and feed it into the GCN model so that the node features can compute themselves again during the aggregation of node features, increasing the difference of features between different nodes. Three 1-D convolutional neural networks with different convolutional kernel depths extract further correlations between node features, and fully connected layers are used for the final classification. Our experimental results show that our approach has an accuracy of 98.28%; therefore, our approach is effective. The contributions of this paper are as follows:

- The function call graph is a static feature that is often used because it captures the intent and behavior features of the function very well. We propose a subgraph extraction approach that effectively removes nonsensical nodes and maximizes the retention of node features. The function call subgraph avoids the interference of nonsense nodes and reduces the dimensionality of the adjacency matrix and feature matrix of the function call graph;
- We extracted the latest API protection level mapping relationship from the Android Open Source Project [11] instead of directly using the one in Pscout [12]. Based on the API protection level relationship, we proposed a function weight feature and demonstrated through experiments that embedding this feature into nodes can effectively help neural networks identify and detect Android malware;
- Since the model learning of GCNs is based on the features of low-pass filters, the node features in the graph structure will converge to similarity during the forward propagation and interfere with our detection. On the one hand, we extract the sensitive function call subgraph. On the other hand, inspired by the GCN formula, we propose an approach of aggregating the features of its nodes one more time to enhance the differences between the different node features;
- Traditional deep learning models cannot learn graph structure type data directly. We propose a GCN + 1-D CNN model using the GCN model to learn the behavior features between different nodes in the function call graph, and we use the 1-D CNN model with different convolutional kernel depths to extract the association relationships between important nodes. The experimental results show that our model has a high accuracy rate in Android malware detection methods.

The rest of the paper is organized as follows: Section 2 describes previous work in Android malware detection. Section 3 is the introduction and implementation process of our approach, including feature extraction, graph extraction, and establishment of the neural network. Section 4 describes the experimental environment, dataset, experimental process, and results. The conclusion and limitations of this paper are presented in Section 5.

## 2. Related Work

In this section, we will introduce the approaches proposed by other researchers for Android malware detection based on static and dynamic features. We describe the methods and models, and we highlight the main contributions and directions for future work. Our research contribution can inspire further questions and future research directions.

### 2.1. Static Features

Features obtained through analysis of the source code or from other information about the application are called static features [3]. Static analysis can detect malware before installation and can be detected in the model environment, thereby reducing the experiment cost.

#### 2.1.1. Traditional Static Features

By decompiling APK files to create file directories, such as assets, AndroidManifest.xml, and classes.dex, researchers can obtain permissions, intents, and other configurations from AndroidManifest.xml and opcodes from classes.dex. They often treat data, such as permissions, intents, and opcodes, as features of the APK, which they feed into a neural network for recognition and classification.

Wu et al. [13] proposed a static feature-based mechanism called DroidMat. They extracted the static features from AndroidManifest.xml, including requested permissions, intent messages passed, components which are regarded as API call entry points, etc. Next, they used many kinds of clustering mechanisms to identify the different intents of the malware to enhance the identification capability of the model. Finally, using the kNN algorithm, they classified the application as benign or malicious. Their method has a better accuracy (97.87%) and recall (87.39%) than the well-known tool Androguard, and it takes half the time of Androguard to analyze the same number of samples.

Li et al. [14] extracted exhaustive features from the application and classified them into eight categories: hardware components, requested permissions, app components, filtered intents, restricted API calls, used permissions, suspicious API calls, and network addresses, although not all of these exhaustive features are meaningful for Android malware detection. Then, they used the principal components analysis (PCA) to choose the more important features. In contrast to traditional machine learning models with shallow structures, such as SVM, they chose a deep learning model with more than three layers, a deep neural network (DNN) as the detection model. They outperformed other machine learning methods with more detailed results, achieving a 97.16% precision. In the future, they will consider combining static and dynamic features to characterize Android applications.

#### 2.1.2. Function Call Graph

The function call graph (FCG) belongs to a kind of static feature. It can capture the semantic information of the function, which the permission-based static analysis method cannot.

Liu et al. [15] proposed G features using information from an FCG. They fed the G features into the machine learning algorithm to detect malware. Their method achieved an 86.9% accuracy in an up-to-date malware testing dataset and avoided collapsing issues induced by the high-dimension vectors of the traditional FCG. Fan et al. [16] proposed an approach to detect Android piggybacked apps called DAPASA. They extracted sensitive subgraphs (SSGs) using the tf–idf-like algorithm that could profile the most suspicious behavior of an app. They extracted five features from the SSG to depict the invocation patterns and fed them into the machine learning algorithms to detect whether the app was piggybacked or benign. Their approach achieved a 94.32% accuracy with only five numeric features. It complemented the permission-based and API-based approaches by combining their proposed five features from a new perspective of the invocation structure. Moreover, their work can be improved upon by building a more detailed behavior model.

For the graph structure features, the original deep learning model cannot directly learn features. It needs to convert the graph structure data into vectors, while the graph neural network can directly learn the features. Feng et al. [17] constructed the approximate call graph from function invocation relationships to represent the app. They extracted each intra-function attributes as node features in the graph. They were then using a graph neural network model to generate a vector representation. Their approach constructs traditional static features into novel graph structure data and does not propose new features based on the graph-structured data. Vinayaka et al. [18] captured the caller–callee relationships between the function to form the function call graph. They considered the difference in the number of nodes in the function call graph due to the difference in APK file size, and they proposed a balanced technique to make the number of nodes similar. Furthermore, they tested five different GCN algorithms to evaluate the method’s performance. Experiments were conducted to compare the performance of the different algorithmic models. The optimal algorithm model they tested achieved a 92.29% accuracy and is also used in this paper. Cai et al. [19] used function calls to learn an app’s behavior features. They proposed enhanced function call graph (E-FCG) to characterize the runtime behaviors of the app and developed a GCN-based algorithm to obtain vector representations of E-FCGs. Their approach overcomes the inability to understand the behavioral characteristics of the app due to missing function properties and the inability of traditional machine learning methods to learn the graphical representations directly.

### 2.2. Dynamic Features

When an Android application is run on a real device or emulator, the runtime behavior features obtained are called dynamic features for monitoring network traffic, battery usage, CPU utilization, requests, and calls among others [3].

Garg et al. [20] proposed the network-based detection app model. They extracted four different traffic categories of network features from each app running on the mobile device, namely, DNS, HTTP, TCP, and origin–destination, and used machine learning classifier algorithms to monitor and learn the network behavior of different apps. Their approach can (1) detect malicious apps using network traces, (2) work with different versions of operating systems, (3) detect unknown apps, and (4) detect infected apps with encrypted data. Their future work will focus on improving the detection rate of unknown applications. Existing dynamic analysis methods rely heavily on characterizing system calls, and these methods are susceptible to system call obfuscation.

Cai et al. [21] proposed a dynamic app classification technique called DroidCat to complement existing methods. By using a diverse set of dynamic features based on method calls and inter-component communication (ICC) intents while fully handling reflection, DroidCat achieves superior robustness than static features, as well as dynamic features relying on system calls. They designed the effects of DroidCat effectiveness under different conditions and the most important dynamic features. They found that features capturing an app execution structure are much more important than typical security features, such as sensitive flows. Their detection technique achieved a 97% F1 measure consistent accuracy for classifying apps evolving over the nine years.

John et al. [22] took the system calls as features representing the operating system’s interaction, and proposed a detection mechanism using GCNs, which uses centrality measures of the system call graph as input features. They are the first application of GCNs for dynamic Android malware detection, achieving a 92.3% accuracy, and they have attempted to combine dynamic features with graph neural network models. Taheri et al. [23] proposed a hybrid feature-based detection method using permissions and intents as static features, and 77 network flows by appending extracted n-gram sequential relations of API calls as 78 dynamic features to detect and classify malware. This approach is the second part of their contribution. In the first part, they presented the CICMalDroid open accessible dataset and labeled its features. This dataset is also used in this paper. They have planned, in the future, to generate an Android dataset with more captured features with a massive sample size.

A comparison of the approaches proposed by different researchers and their contributions is shown in Table 1. Compared with the permission-based static features and dynamic features, the function call graph with node features can leverage topology information to infer apps’ behavior features. Although code obfuscation affects static feature analysis, the function renaming cannot change the topology of the function call graph. It can reduce the impact of the function renaming, and it also avoids the problems, such as high experimentation cost of dynamic features and difficulty in triggering the full malicious behavior of the application. While using the graph neural network model can make better and more extensive use of graph structure type data, in this paper, we use the function call graph of static features to represent APK files and GCNs to learn graph structure data. Our proposed approach solves the drawbacks caused by function call graphs and GCN models to a certain extent and proves the effectiveness of our method through experiments.

## 3. Method

**Figure 1:**
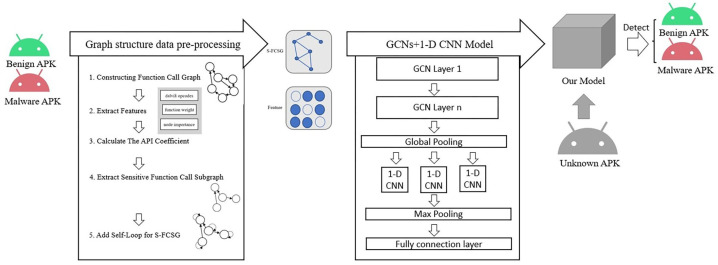
Overall framework of our approach.

In this section, we will focus on the proposed API-based node feature function weight, the subgraph extraction method, and the role of adding node self-loops. As illustrated in Figure 1, our model is the overall framework of our approach. The APK samples of known categories are first processed by graph structure data pre-processing to generate the function call graph required by the model and their corresponding node feature matrices. Then, the function call graph and node feature matrices are fed into GCN and 1-D CNN models. Following the forward and backward propagation of the model, the parameters of the model are updated and the APK samples are classified. The process of graph structure data pre-processing and model detection is divided into different phases. These phases are explained in this section, and the corresponding subsections have been marked in the figure.

### 3.1. Feature Extractor

Our approach extracts three types of features from the FCG and decompiled APK files. Two of these features are Dalvik opcodes and function weight, which are obtained from each function in the DEX file, and the remaining one is the node importance based on the graph structure level. Finally, we concatenate the three types of features as the node feature of the FCG.

#### 3.1.1. Dalvik Opcodes

Dalvik is a virtual machine especially designed by Google for the Android operating system, and opcodes exist in the code of the DEX file. According to the opcode list provided by Gabor Paller [24], we extract the Dalvik opcodes from each method in the class and classify their variety as our first feature. Prior to commencing our work, we need to distinguish between internal and external classes. The internal class is obtained from the DEX file of the decompiled APK. We can obtain the code sources of each internal class, but the external class cannot. They stem from third-party libraries. Due to their difference, we are using an array with a length of 14 to represent the Dalvik opcode feature of each method.

For the external class, it cannot obtain the detailed code instructions from each function, so we represent the function of the external class as dalvik_opcodes[0] = 1.

For the internal class, we obtain the Dalvik opcodes by analyzing each instruction in each function of the internal class. In order to better analyze these instructions in each function, divide them into 13 categories, as shown in Table 2. Then, obtain the corresponding keyword and *i* value according to the Dalvik instruction opcodes and store as dalvik_opcodes[*i*] = 1.

For each method in the class of the DEX file, we represent the Dalvik opcode feature of each method as dalvik_opcodes[*i*] = 1 (i∈[0,13]).

Note that if dalvik_opcodes[0] = 1, then dalvik_opcodes[*i*] (i∈[1,12]) could equal 0. The converse is also true. The dalvik_opcodes is an array of variables defined in Python, with a total length of 14 used to store the state of opcodes at different index *i*.

#### 3.1.2. Node Importance

The FCG is a directed graph that is extracted from decompiled APK files. In a directed graph, the in-degree and out-degree of each node are different, while the degree values can directly reflect the importance of that node. We can analyze the current node’s behavior features based on a node’s in-degree and out-degree. Generally, we use only in-degree for external class function nodes and only out-degree for app initialization function nodes.

In graph theory and network analysis, centrality is an indicator that judges the influence of nodes in the network. The higher the degree of a node, the higher the degree centrality of the node, which means that the node has more influence in the network. This is the degree centrality [25]. The calculation formula is as Equation (1).

(1) $$Degree\;\;Centrality=\frac{N_{indegree}+N_{outdegree}}{n-1}$$

Although degree centrality reflects the influence of a node and includes the in-degree and out-degree of nodes, we cannot use this metric to find out whether the node is frequently pointed to other nodes or not. We need the node’s in-degree and out-degree values to analyze whether the node plays the role of initiator, executor, or intermediate node in the network. Therefore, we combine the in-degree and out-degree of the node and degree centrality as the feature of each node in the FCG, represented as

NodeImportance=[InDegree,OutDegree,DegreeCentrality]

#### 3.1.3. Function Weight

Application programming interfaces (APIs) are some pre-defined functions. Through APIs, functions can be rapidly expanded for applications without understanding how they are implemented to improve development efficiency.

The programmers often use APIs to develop Android applications. They can access key information from the smartphone via the APIs, but using the APIs requires configuring the permission in AndroidManifest.xml, such as android.permission. READ_SMS permission [26] allows for applications to read SMS messages. While each permission has its protection levels, the permission mentioned above is dangerous, which means that it has higher risk permissions, which allow the application requesting authorization to access the user’s private data or gain control of the device that can adversely affect the user. We make the maps between APIs and protection levels. Then, we can analyze the code in the DEX file to obtain the permission protection levels required by the application and calculate the weight of each API function as each node feature of the FCG.

To create the maps between APIs and protection levels, we need to map between the APIs and required permissions, and between the permissions and protection levels first. As for the maps between permissions and protection levels, they can be obtained from the AndroidManifest.xml of the AOSP, while for the maps between APIs and permissions, some use Pscout to build the maps [12]. We think that Pscout is complete but outdated because its latest maps are based on Android 5.1 released in 2018, and this version has since been replaced by several generations (the latest Android system is Android 13, which was launched on 12 May 2022).

Google has formally documented permission specifications in two ways since Android 6.0 (API level 23) [27]:

- Using Java annotation @requiresPermission to associate APIs with permissions;
- Using @link android.Manifest.permission# to describe an API’s required permissions.

**Figure 2:**
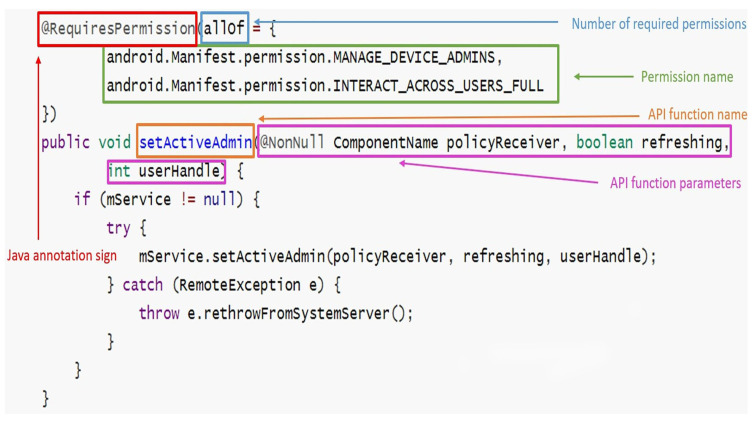
Google formally documents permission specifications using @RequiresPermission annotation in the AOSP.

**Figure 3:**
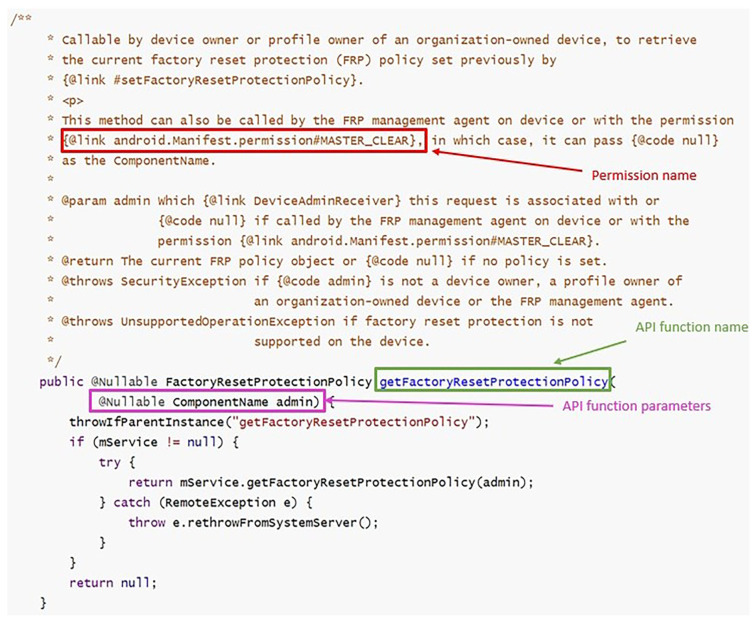
Google formally documents permission specifications using {@link android.Manifest.permission#XXX} annotation in the AOSP.

In the two ways mentioned above, we can extract the permission corresponding to the API from the Android Open Source Project (AOSP) [11] and form the API permission mapping. As shown in Figure 2, this is a Java code fragment from the AOSP. This code fragment could tell us that the setActiveAdmin API needs MANAGE_DEVICE_ADMINS and INTERACT_ACROSS_USERS_FULL permission. Similarly, Figure 3 tells us that the getFactoryResetProtectionPolicy API needs MASTER_CLEAR permission. As shown in Table 3, this is the explanation of the fields we extracted from Figure 2 and Figure 3. A total of 1640 APIs were extracted from the AOSP.

We use the extracted permissions protection level mapping and the API permission mapping to build the maps between APIs and protection levels.

According to the counts of the different APIs called, we calculate the API call weight of each function. If the API permission has multiple protection levels, only the maximum weight value of the protection level is taken for the calculation. For each function in the internal classes, we represent the API call weight of each function as Equation (2). In contrast, for functions in the external classes, we represent the API call weight of each method as the weight value of the protection level of selves, as defined by Equation (3). Normal, signature, privileged, and dangerous are the four common levels of protection for APIs. As the normal permission is the default value for API permissions, it is low-risk. The system will automatically grant such permissions to the application without explicit permission from the user. All of the protection levels, except the normal protection level, carry some risk, and we need to capture both the identified and the potential risks fully. Therefore, we define the API weight for the normal permission as 0 and the other permissions as 1. We use this method to calculate the function weight of 12,898 APK files (6530 benign APK files and 6368 malicious files) in our dataset, obtained from open accessible datasets. Detailed information on the datasets used in our work can be found in Section 4.2.

(2) $$\begin{array}{c} API\_Call\_Weight_{internal\_method}=\sum^{n}weight_{protection\;level} \end{array}$$

(3) $$API\_Call\_Weight_{external\_method}=\max\left(weight_{API}\right)$$

If a function has a higher value of API call weight, it means that there are more API calls in this function. However, there are more API calls at other protection levels than at dangerous protection levels, which can only mean more behavior exists in this function. Therefore, we put forward the ratio between API call counts with a dangerous protection level and the total API call counts defined by Equation (4). This ratio represents the percentage of calls to APIs identified as dangerous.

(4) $$ratio_{each\_method}=\frac{dangerous\_api\_call\_counts}{api\_call\_counts}$$

We select the API call weight, ratio, and their product as the function weight feature of each node in the FCG represented as

$$method\;weight_{each\_method}=\left[api\_call\_weight,\;ratio,\;api\_call\_weight*ratio\right]$$

Furthermore, we have made the following assessment on these features:

Assessment 1: Randomly select 2000 APK files from our dataset.

**Figure 4:**
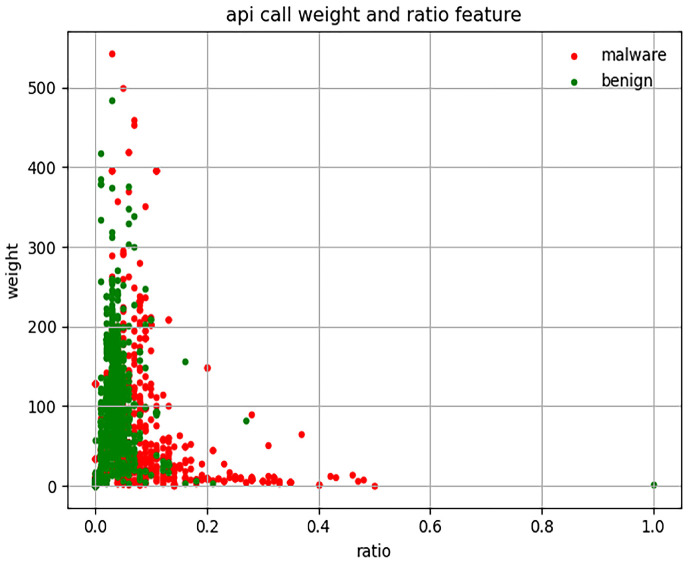
API call weight and the ratio of 2000 APK files.

In Assessment 1, we calculate the sum of the API call weight and the ratio of 2000 APK files (1000 malware and 1000 benign), as shown in Figure 4. In general, the larger the API call weight value, the larger the APK file. Although the size of 2000 randomly selected APK files varies greatly, we can see that under the same weight value, the ratio value of malware is greater than that of benign, which means that the API with dangerous protection permission in this APK file is called more frequently. In the following assessment, we will select samples with similar sizes from these samples to demonstrate this finding.

Assessment 2: Select 300 APK files of similar size in Assessment 1.

**Figure 5:**
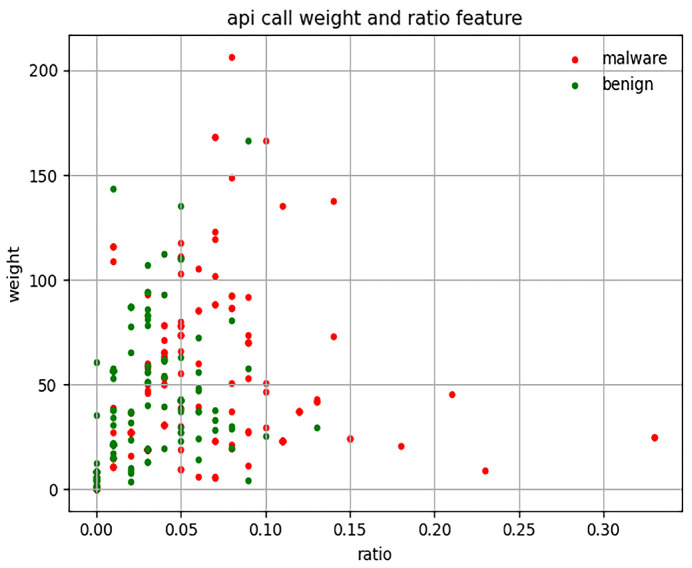
API call weight and ratio of 300 APK files of similar size.

In Assessment 2, we find 300 samples (150 malware and 150 benign) of similar size from 2000 samples and calculate the sum of the API call weight and the ratio of those samples, as shown in Figure 5. Normally, In the case of the same weight value, the ratio value of malware is greater than benign, and in the case of the same rational value, the weight value of malware is greater than benign. Therefore, malware’s weight value and ratio value are generally greater than benign.

As we can see in Figure 4 and Figure 5, malware is more distributed on the upper right side of the scatter plot than benign. According to the results of Assessments 1 and 2, we speculate that the value of the product between the weight and ratio of malware should generally be greater than benign. Hence, we conduct Assessments 3 and 4.

Assessment 3: Calculate the value of weight∗ratio based on Assessment 2.

**Figure 6:**
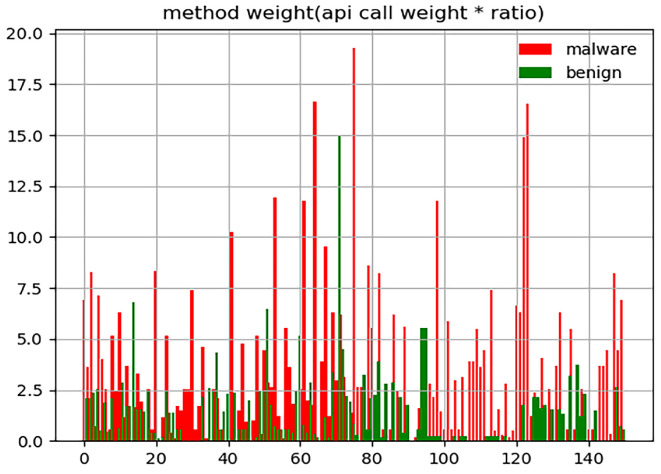
The value of the API call weight∗ratio of 300 APK files of similar size.

As shown in Figure 6, it can basically prove our above speculation.

Assessment 4: Select 100 APK files of similar size in Assessment 3 to calculate the value of weight∗ratio.

**Figure 7:**
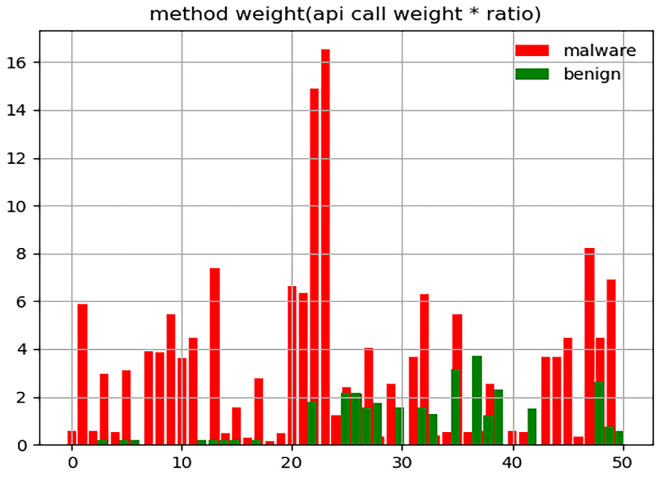
The value of the API call weight∗ratio of 100 APK files of similar size.

In order to obtain our inference more clearly from the figure, we calculated the value of weight∗ratio again after reducing the number of APK samples. As shown in Figure 7, we can definitely prove our speculation: the value of the weight∗ratio of malware should generally be greater than benign.

With the four assessments above, the API call weight in function weight can reflect the size of an APK file to some extent. We can clearly find that malware’s ratio, the weight∗ratio value is generally larger than benign’s ratio for a similar API call weight. Thus, even without precise computation by the neural network, we can use this feature to distinguish the APK class roughly. The later experimental sections show that using this node feature makes our model very effective.

### 3.2. Graph Extractor

In this Section, our approach first extracts the function call graph (FCG) and, on top of the FCG, extracts the sensitive function call subgraph (S-FCSG). The extraction of the S-FCSG involves three steps: calculate the API coefficient, extract the FCSG, and extract the S-FCSG.

#### 3.2.1. Generate an Entire Function Call Graph (FCG)

**Figure 8:**
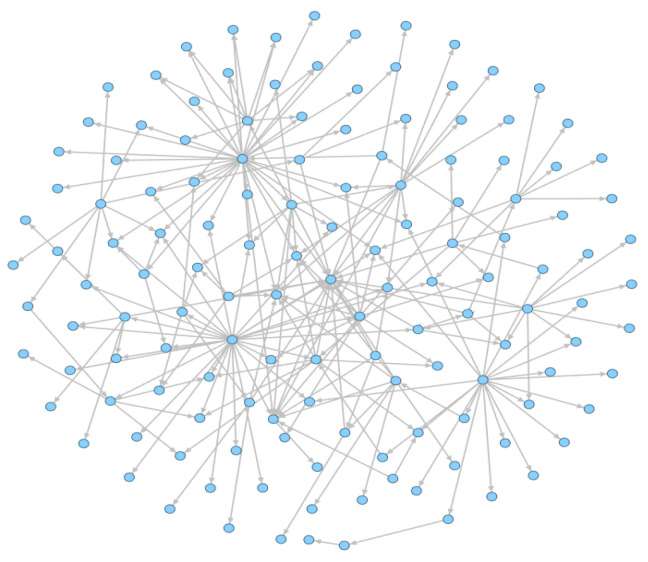
The FCG of 382faa3f0119848f4c0b325fe50ae8659be2b09e069f6660436276358856708b of the Drebin dataset. The nodes represent the functions of the APK file. The edges represent the call–callee relationship between the different functions.

Androguard [28] is a complete Python tool used to play with Android files and is used to extract the FCG in our work. It can decompile the APK file and obtain the functions in the class. By analyzing the invoked instructions in each function, it takes the calling and called functions as nodes, adds directed edges according to the calling relationship, and then builds a directed function call graph. The function call graph can automatically capture their behavior features through this call–callee relationship between different functions. As shown in Figure 8 from this directed graph, we can discover the call–callee relationships between the different nodes.

#### 3.2.2. Calculate the API Coefficient

The API coefficient is calculated to indicate the importance of APIs in Android applications. If only the frequency of API occurrences in the dataset is calculated as the API coefficient, then the measurement will be biased, such as MIGDroid [29].

Inspired by the idea of the TF–IDF [30] algorithm, we apply the TF–IDF algorithm suitable for our work to calculate the API coefficient, and use the API coefficient to express the importance of this API. From the 1640 APIs extracted from the AOSP above, we select 978 APIs with high protection levels and a special call frequency, and use these 978 APIs to build a sensitive API set.

We have defined four terms to help us calculate the API coefficient.

- $count(api_{i},apk)$: the counts of $api_{i}$ is called in the APK;
- count(apk,∗): the total counts of API called in the APK;
- number(c): the counts of APKs of type *c* in the dataset. *c* indicates whether the category of the APK is malicious or benign;
- $number(api_{i})$: the counts of APK which are called $api_{i}$.

According to the formulas in TF–IDF, we propose the formulas applicable to our work, as follows:

(5) $$TF\left(api_{i},apk\right)=\frac{count(api_{i},apk)}{count(apk,*)}$$

(6) $$IDF\left(api_{i},c\right)=\log\frac{number(c)}{number(api_{i})+1}$$

(7) $$TF-IDF\left(c\right)=TF\left(api_{i},apk\right)*IDF\left(api_{i},c\right)$$

TF−IDF(malware) indicates the TF–IDF value of malware.

TF−IDF(benign) indicates the TF–IDF value of benign.

To avoid program exceptions with denominator 0 caused by unused APIs, we added 1 to the denominator of the log function in the IDF formula. The API coefficient value should be proportional to the value of TF−IDF(malware) and TF−IDF(benign). The calculation formula is as follows:

(8) $$API\;coefficient\left(api_{i}\right)=TF-IDF\left(malware\right)*TF-IDF\left(benign\right)$$

Through calculation, we can learn that in our sensitive API set, the API coefficient value of getInstance(context) ranks first and its TF and TF–IDF values in malware and benign also rank first, indicating that this API has been called the most times and is the most important. Several APIs with a dangerous protection level also rank high, such as connect(WifiP2pManager$Channel,WifiP2pConfig,WifiP2pManager$ActionListener), getExternalStorageDirectory(), etc.

#### 3.2.3. Extract the Function Call Subgraph (FCSG)

We extract the function call subgraph (FCSG) containing all sensitive API nodes based on the sensitive API set. As shown in Algorithm 1, this is our extraction process.
**Algorithm 1** Generate the Function Call Subgraph (FCSG)**Input:** 
FCG = {*V*,*E*}; SENSITIVE_API_SET;**Output:** 
FCSG1: SUBGRAPH_NODE_LIST←Φ2: **for** each  $V_{api}\;\in \;SENSITIVE\_API\_SET$ **do**3:     **if** $V_{api}\in FCG$ **then**4:          **for** each $V_{i}\in FCG$ **do**5:              **if** $shortest\_path(V_{i},V_{api})<=2$ **then**6:                  $SUBGRAPH\_NODE\_LIST\leftarrow V_{api}\cup \left\{V_{i}\right\}$7:              **end if**8:          **end for**9:     **end if**10:**end for**11:FCSG←subgraph(FCG,SUBGRAPH_NODE_LIST)12:**return ** FCSG

Algorithm 1 shows the step of extracting the FCSG with the input of the FCG of each application and sensitive API set, which is extracted from the AOSP we mentioned earlier. We define SBUGRAPH_NODE_LIST variable to save API nodes and their neighbor nodes. Note that the FCG is considered an undirected graph during the extraction of the FCSG.

In Algorithm 1, the function of $shortest\_path(V_{i},V_{api})$ is used to calculate the shortest distance between $V_{i}$ and $V_{api}$ vertices, set(SBUGRAPH_NODE_LIST) function is for removing duplicate vertices in the list. subgraph(FCG,SBUGRAPH_NODE_LIST) function extracts the subgraph FCSG of the FCG according to the nodes in SBUGRAPH_NODE_LIST.

**Figure 9:**
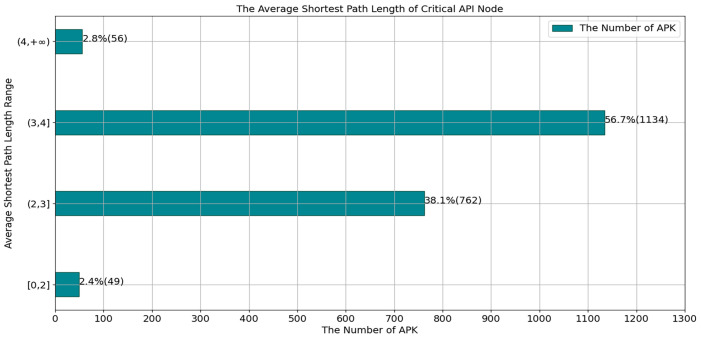
Average shortest path length in the 2001 randomly selected APK files from the dataset.

In the function of $shortest\_path(V_{i},V_{api})$, it extracts neighbor nodes with the shortest path length that is less than or equal to 2. For the reason that we randomly selected 2001 APK files from the dataset, it turns out that the average shortest path length of API nodes to its neighbor nodes ranges from the greatest amount (94.8%) of APKs at (2, 4) and only a few APKs are greater than 4, as shown in Figure 9. To extract subgraphs, we set the neighboring nodes with the shortest path length of less than or equal to 2.

#### 3.2.4. Generate the Sensitive Function Call Subgraph (S-FCSG)

**Figure 10:**
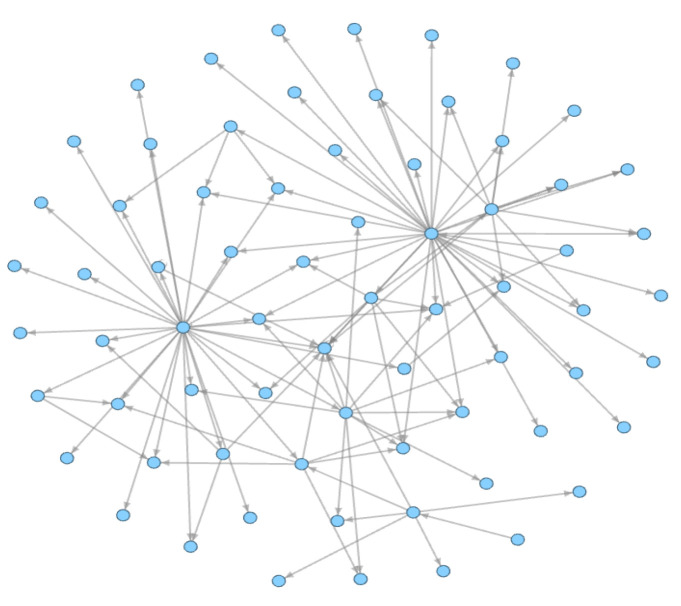
The S-FCSG of 382faa3f0119848f4c0b325fe50ae8659be2b09e069f6660436276358856708b of the Drebin dataset. The meanings of the nodes and edges in the figure are the same as those in Figure 8.

We further extract the sensitive function call subgraph (S-FCSG) from the FCSG. As shown in Figure 10, this is an S-FCSG, and Algorithm 2 is our extraction process.
**Algorithm 2** Generate the Sensitive Function Call Subgraph (S-FCSG)**Input:** 
FCSG = {*V*,*E*}; SENSITIVE_API_SET; API_COEFFICIENT_LIST**Output:** 
S−FCSG1:SBUGRAPH_NODE_LIST←ϕ2:**for** each  $V_{i}\;\in \;FCSG$ **do**3:   **if** $V_{i}\in SENSITIVE\_API\_SET$ **and**   $coefficient\_rank(V_{i},API\_COEFFICIENT\_LIST)$ **then**4:       $V_{n}\leftarrow bfs\_tree\left(FCSG,V_{i}\right)$5:   **end if**6:**end for**7:$SBUGRAPH\_NODE\_LIST\leftarrow \{V_{n}\}$8:S−FCSG←subgraph(FCSG,SBUGRAPH_NODE_LIST)9:**return **S−FCSG

Algorithm 2 shows the extraction process of the S-FCSG using the FCSG, the sensitive API set, and the API coefficient list as input. The algorithm’s essence is to extract the sensitive subgraph with the high API coefficient nodes from the FCSG. The FCSG is also considered an undirected graph during the extraction.

The function of the variable SBUGRAPH_NODE_LIST is the same as that of the variable SBUGRAPH_NODE_LIST in Algorithm 1.

The $coefficient\_rank(V_{i},API\_COEFFICIENT\_LIST)$ function is used to calculate the rank of the API coefficient of API node $V_{i}$, the function of $bfs\_tree(FCSG,V_{i})$ utilizes breadth-first search to find all connected neighbor nodes of $V_{i}$ in the FCSG.

In the process of subgraph extraction, we treat directed graphs as undirected graphs using the all-preserving approach. We randomly tested 3000 sample APKs in the dataset, extracted subgraphs starting from the top 1, 3, 5, 7, 10, 20, and 30 sensitive API nodes sorted by API coefficients, and observed the changes in the number of nodes and node features in the graph, respectively, as shown in Table 4. The subgraphs extracted using this approach may contain API nodes that do not have a high API coefficient ranking, which are API nodes within two hops of the top API coefficient ranking node.

With Table 4, the S-FCSG effectively reduces the number of nodes in the function call graph, directly reducing the complexity of the graph structure while retaining the maximum node features and structure. Based on the characteristics of the graph neural networks, the S-FCSG avoids the tendency for high feature weight nodes to be similar to nonsensical nodes or less important nodes during the propagation of the graph convolutional neural network, thus reducing the interference of redundant nodes in our model learning. The dimensionality of the adjacency and feature matrices of the S-FCSG is smaller than that of the FCG, which speeds up the training of our model below and consumes resources. Following the comparison, we chose the S-FCSG with the top 10 parameters for the next experiments.

### 3.3. Neural Network Model

**Figure 11:**
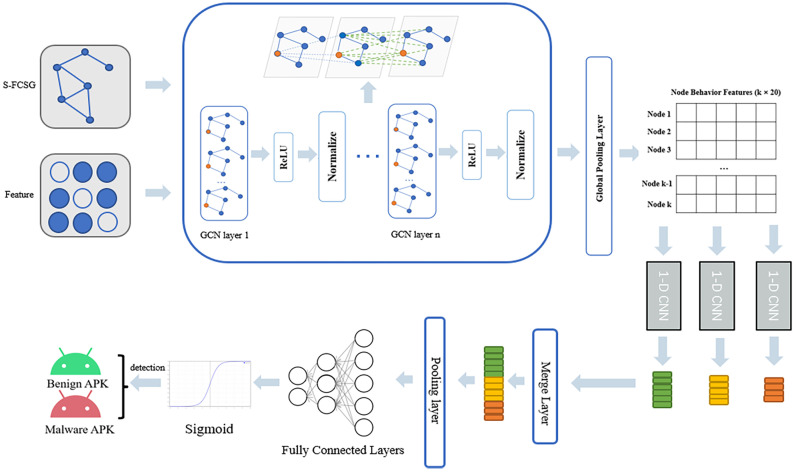
Our GCN+1-D CNN model.

In this section, we hope to use a neural network model to automatically capture the semantic relationship between different nodes in the graph structure. In this paper, we propose a combined GCN+1-D CNN model, as shown in Figure 11. The GCN model is used to embed the graph structure data and node features directly. During the propagation of the GCN model, it is possible to aggregate the features of the current node and its neighbors, allowing for feature learning between different nodes. A 1-D CNN model is used to further capture the correlation between the node features, which after learning by the GCN model, then feeds the captured relevant features back to the fully connected network for classification.

#### 3.3.1. Graph Convolutional Networks

For the GCN model, we compare two algorithmic models:

(1) GraphConv [31]:

Kipf et al. consider a layer-wise propagation rule of a multi-layer graph convolutional network (GCN), as shown in Equation (9):

(9) $$H^{\left(l+1\right)}=\sigma \left({\tilde{D}}^{-\frac{1}{2}}\tilde{A}{\tilde{D}}^{-\frac{1}{2}}H^{\left(l\right)}W^{\left(l\right)}\right)$$

$\tilde{A}$ is an adjacency matrix with an added identity matrix that is pooling the information of each node and its neighbors. $W^{\left(l\right)}$ is a learnable weight matrix and σ is an activation function.

**Figure 12:**
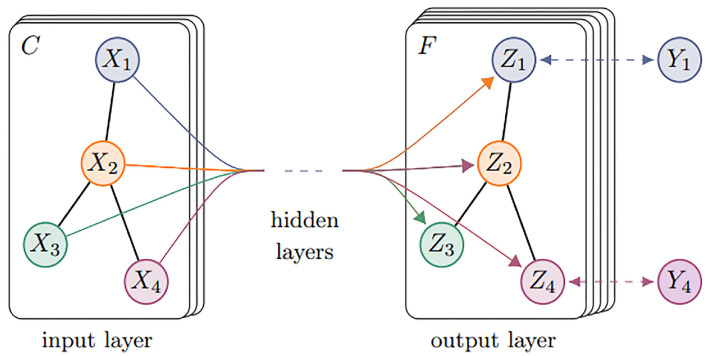
Schematic depiction of the multi-layer graph convolutional network (GCN) [31].

Equation (10) is the simple form of the forward propagation model and Figure 12 is the schematic depiction of the multi-layer graph convolutional network (GCN) for semi-supervised learning.

(10) $$Z=f\left(X,A\right)=softmax\left(\hat{A}\;ReLu\left(\hat{A}XW^{\left(0\right)}\right)W^{\left(1\right)}\right),\;\hat{A}={\tilde{D}}^{-\frac{1}{2}}\tilde{A}{\tilde{D}}^{-\frac{1}{2}}$$

**Figure 13:**
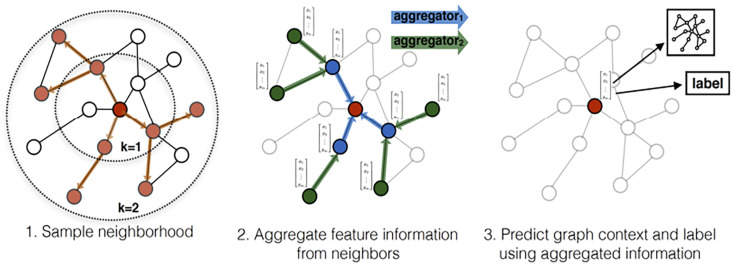
Visual illustration of the GraphSAGE sample and aggregate approach [32].

(2) GraphSAGE [32]:

The GraphSAGE approach is closely related to GraphConv. Nodes aggregate features from their neighbors and gain more and more features from further neighbor nodes of the graph with the increase of GraphSAGE layers. Equations (11) and (12) are the process of aggregation features. Equation (13) is the L2 normalization. Figure 13 visually illustrates the GraphSAGE sample and aggregate approach.

(11) $$h_{N(i)}^{\left(l+1\right)}=AGGREGATE\left(h_{j}^{l},\forall j\in N\left(i\right)\right)$$

(12) $$h_{i}^{\left(l+1\right)}=\sigma \left(W*concat\left(h_{i}^{\left(l\right)},h_{N(i)}^{\left(l+1\right)}\right)\right)$$

(13) $$h_{i}^{\left(l+1\right)}=\frac{h_{i}^{\left(l+1\right)}}{{∥h_{i}^{\left(l+1\right)}∥}_{2}}$$

In general, the low-pass filter in the GNN mainly retains the commonality of node features. It inevitably ignores differences so that the learned feature representations of connected nodes are similar [10]. Similarly, the GraphConv and GraphSAGE models mentioned in the paper make the feature representations of the interconnected nodes converge to be similar in the forward propagation of the models. In Section 3.2, we extract sensitive function call subgraphs and remove nonsensical nodes and less significant nodes from the graph, which can effectively avoid the feature representation of important nodes that are similar to those of nonsensical nodes after the process of forward propagation of the GCN model.

**Figure 14:**
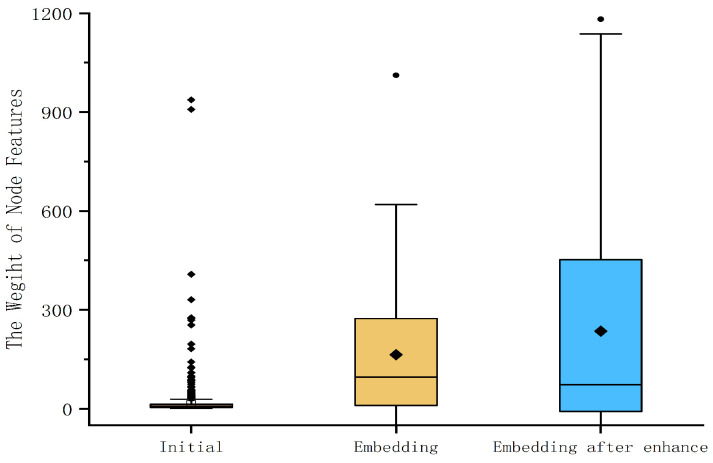
Comparison of the feature weights of com.moyou.gjqx.uc.apk of the Androzoo dataset.

Compared to the algorithm of GrapConv, GraphSAGE is an inductive graph embedding that can embed nodes that never appeared, but GraphConv cannot. Therefore, we take GrapSAGE as our GCN model and set the parameters AGGREGATE to mean and σ to ReLu. Influenced by Equation (9) in GraphConv, we added the self-loop to the S-FCSG before feeding it into the GCN model in order to allow the GraphSAGE model to aggregate its own features once more as it propagates the aggregated node features forward, further enhancing the features of the important nodes on the basis of preventing them from being similar to the features of the nonsensical nodes. As shown in Figure 14, this shows the feature weights initially after the GraphSAGE model embedding and after the GraphSAGE with the self-loop S-FCSG model embedding, respectively. We set both the weight and bias in the model to none during this process.

#### 3.3.2. Global Pooling Layer

The S-FCSG has a high-dimensional adjacency matrix and node feature matrix. We use the global pooling layer to extract the critical features and reduce the dimensionality of the node feature matrix. The sort pooling model [33] is used as our global pooling layer. It first sorts the node features in ascending order along the feature dimension of the GCN model output and selects the sorted feature weight of top-K nodes. The nodal feature matrices of different dimensions can all be normalized to the same dimension after the global pooling layer, which facilitates the definition and calculation of our next model. We combine the extracted K node feature vectors into a K-dimensional nodal feature matrix.

The size of K represents the number of significant nodes selected for our experiment, and we experimentally demonstrate in the next Section that using different values of K in the global pooling layer gives different experimental results.

#### 3.3.3. The 1-D Convolution Neural Networks and Fully Connected Layer

Our 1-D convolutional neural network and fully connected layer consist of three 1-D convolutional layers, a merge layer, a max pooling layer, and a fully connected layer. Each convolutional layer has a different convolutional kernel depth, which is used to extract behavior features between nodes at different granularities. The merge layer joins the three node feature vectors from the 1-D CNN output, and we set the sampling kernel as three and stride as three in the next max pooling layer. Then, the fully connected layers have 128 hidden layer nodes. Finally, we use the sigmoid function for the binary classification.

## 4. Experiment and Evaluation

### 4.1. Experimental Software and Environment

Pytorch 1.10 was used to build our model framework and Androguard [28] was used to extract the function call graph from APK. DGL [34] was used to implement the GraphSAGE model, and Joblib [35] was used to help us, in parallel, decompile the APK. The environment was established on Windows 10 with 8GB of RAM for analysis and extraction, and Ubuntu 18.04 with 11GB of VRAM was used for calculation. Both operating systems had installed Python 3.7.

### 4.2. Dataset

In our work, we used 6530 benign APKs that were generated after 1 Jan 2017 from the Androzoo [36] dataset as our benign dataset, and 6368 malware APKs from Drebin [37] and CICMalDroid 2020 [38] were taken as our malware dataset, 3500 and 2868, respectively. However, some of these APKs were broken, and we cleaned up some APKs that could not be decompiled or from which we could not obtain code resources. The dataset was divided into 80% training set and 20% verification set.

In parallel, we decompiled these APKs in advance and utilized the pickle method of Python to form serialized files, which can greatly reduce the time for analyzing APK files during training.

### 4.3. Evaluation Indicator

We evaluated our model using standard metrics, such as accuracy, precision, F1 score, TPR, FPR, and AUC. These metrics were calculated as Equation $Accuracy=\frac{\left(TP+TN\right)}{TP+TN+FP+FN)}$, $Precision=\frac{TP}{\left(TP+FP\right)}$, $F1\;Score=\frac{2*Precision*Recall}{\left(Precision+Recall\right)}$, $TPR=\frac{TP}{\left(TP+FN\right)}$, $FPR=\frac{FP}{\left(FP+TN\right)}$, and AUC is the area under ROC curve. In the above equations, TP denotes the counts of malware correctly detected, FP denotes the counts of benign APKs incorrectly classified as malicious, TN denotes the counts of benign APKs correctly detected, and FN denotes the counts of malware APKs incorrectly classified as benign.

### 4.4. Experimental Process and Result

**Figure 15:**
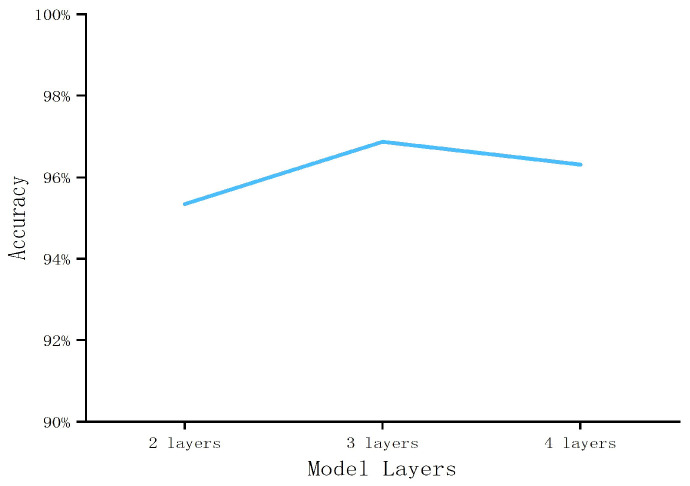
Experimental results for the different GCN model layers (K = 20).

During our experiments, there were many parameters that influenced the outcome of our experiments. The first was the number of layers of the GCN model. We started our experiments with a two-layer GCN model because of the recommendation made by Hamilton et al. [32]. The different node features in the S-FCSG gradually converged to be similar to the GCN model that was continuously trained. This does not mean that more layers in the GCN model were better in our approach. We tested the effect of the GCN model with two, three, and four layers on our experimental results. We observed that the detection accuracy of the model with three layers was higher at K = 20, as shown in Figure 15.

In addition to the effect of the number of model layers, the size of the K-value in the global pooling layer also affected our experimental results. The size of the K-value represented the dimensionality of the behavior features extracted from the APK file. A higher dimensionality may contain more information about the behavioral features, but may also result in a feature overlap, which is not conducive to learning important behavior features. Then, we tested the effect on our experimental results at different GCN layers with K-values of 20, 40, and 60, respectively. As shown in Table 5, the experimental results use GraphSAGE with a self-loop graph for different numbers of layers and K-values as input.

**Figure 16:**
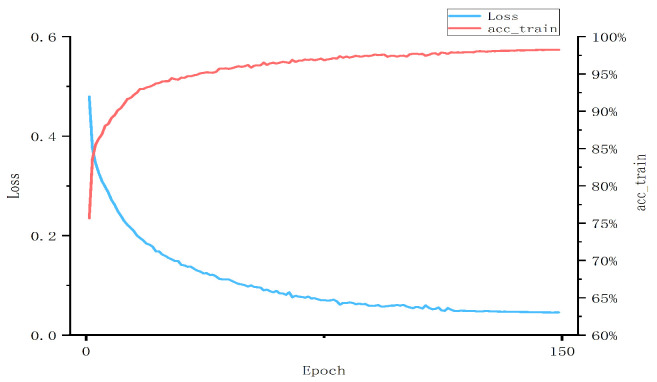
Loss and accuracy of training.

In Table 5, we find that when the number of layers of the GCN model is low, the larger the K-value, i.e., the larger the dimensionality of the node behavior features, the better the results. However, this phenomenon is no longer evident as the number of model layers increases. As the number of layers of the GCN model increases, the accuracy of the model detection is higher at K-values of 20 and 40 than when the number of layers of the GCN model is two. This may be because the node features have not yet fully aggregated the features of neighboring nodes in two layers of the GCN model, and the model cannot correctly identify the APK category at low feature dimensions. However, when the value of K is 60 when the multi-layer GCN model is used again to aggregate node features, the features may overlap, and the features used lose their original importance, causing interference in the model detection and making the final result inferior to that of the two-layer GCN model. By our experimental comparison of different model layers and K-values, the GCN model has the best results in our approach when the number of layers is three and K = 40, with an accuracy of 98.28%. The epoch of our training process is set to 150, as shown in Figure 16 for the experimental process with GCN model layers of three and K of 40. When the epoch reaches 150, the loss function curve and the training accuracy curve have fully converged.

**Figure 17:**
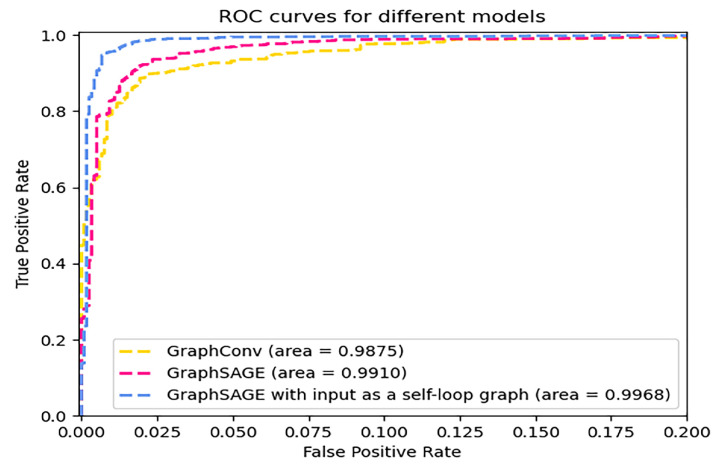
ROC curves for the different models.

Under the same parameters of the model, we compared the results of GraphConv [31], GraphSAGE [32], and GraphSAGE with input as a self-loop graph, as shown in Figure 17. The ROC plot shows that the classification effect of using the GraphSAGE with input as a self-loop graph is higher than the other two models, which shows that we can effectively enhance the important node features by adding self-loop to the S-FCSG and improve the classification performance of the original model.

**Figure 18:**
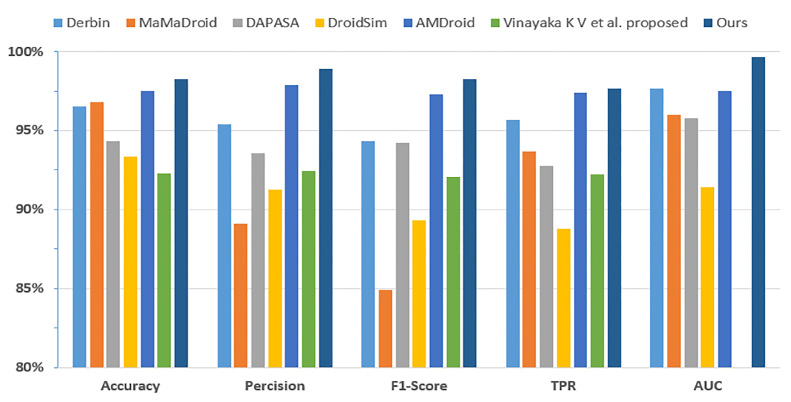
Comparison of our approach with existing approaches.

We compared several existing malware detection methods. The model algorithms ranged from machine learning in the past to the latest GCN models, and the use of features from single permission to complex behavior features in the function call graph. In particular, the approach proposed by Vinayaka et al. [18] uses the FCG as a feature and the GCN model for detection. However, they use the readout function to extract the overall features of nodes in the FCG, which amounts to the aggregation of both important and nonsense node features. Table 6 and Figure 18 compare our approach with the existing ones. The closer the value of AUC is to 1, the better the performance of the classifier. By comparing the size of the AUC area and other metrics, our approach has better results and greater performance in Android malware detection.

## 5. Conclusions

In this paper, we propose an Android malware detection approach to enhance the differences of node features in a function call graph (FCG). Through the validation of the function weight feature proposed in Section 3.1.3, we know that the high permission protection level of APIs is generally more frequent in malicious APKs than in benign APKs. Based on the FCG, we obtained the critical nodes in the graph and extracted them and their neighbors to build the sensitive function call subgraph (S-FCSG). Firstly, the S-FCSG is able to capture important nodes while effectively reducing the number of nodes in the FCG and maximizing the retention of node features and structures. Then, the S-FCSG removes meaningless nodes and it prevents important node features from becoming meaningless during the propagation of the GCN, reducing the impact of low-pass filterability on the interference of important information learned by our model and the effect of the experiment. Based on the GraphSAGE aggregation of node features, we considered involving our node features in the aggregation operation, increasing the weight of important node features and the differences between different node features. The important node features are extracted through the global pooling layer as the node behavior features of the S-FCSG so that the information with significant features can facilitate the identification and detection of our model. Experiments show that our approach is better at identifying and detecting Android malware than previous machine learning-based approaches and current FCG-based approaches.

We have focused on the node features of the graph and the characteristics of the GCN model in this paper, but the graph structure is rich in semantics and there are many entry points to study, such as nodes, edges, attributes, and types of graphs. In the future, we will first study the subgraph extraction method. In our approach, all of the datasets used the same subgraph extraction method. Although this method is effective, we want to adopt a better dynamic subgraph extraction method using the combined GraphSAGE [32] model. Secondly, the important assessment of the nodes in the graph is also advantageous for research content. Our method uses feature weights to evaluate the degree of importance of a node, and a dynamic method of evaluating node importance proposed by Huang et al. [42] is a very novel method worthy of our research. Finally, making the graph richer in feature information will make it easier to identify the model, so the construction of heterogeneous graphs will also be our next research step. In short, malware detection based on graph structure and graph convolutional networks has a lot of space for future study.

## Figures and Tables

**Table 1:** The comparison of different approaches.

Author	Year	Feature	Algorithm Model	Contribution and Future Work
Wu et al. [13]	2012	Static feature (requested permissions, intent messages passed, and component)	kNN algorithm	Takes half the time of Androguard to analyze the same number of samples.
Li et al. [14]	2018	Static feature (hardware components, requested permissions, app components, filtered intents, restricted API calls, used permissions, suspicious API calls, and network addresses)	deep neural network (DNN)	In the future, they will consider combining static and dynamic features to characterize Android applications.
Liu et al. [15]	2018	Static feature (function call graph)	machine learning algorithm	Avoids collapsing issues induced by the high-dimension vectors of traditional FCGs.
Fan et al. [16]	2017	Static feature (sensitive function call subgraph)	machine learning algorithm	Complements permission-based and API-based approaches from a new perspective of the invocation structure.
Feng et al. [17]	2020	Static feature (approximate call graph)	graph neural network (GNN)	Constructed traditional static features into novel graph structure data.
Vinayaka et al. [18]	2021	Static feature (function call graph)	graph convolutional network (GCN)	Proposed a balanced technique to make the number of nodes similar.
Cai et al. [19]	2021	Static feature (enhanced function call graph)	graph convolutional network (GCN)	Overcomes the inability to understand the behavioral characteristics of the app due to missing function properties.
Garg et al. [20]	2017	Dynamic feature (four different traffic categories of network features)	machine learning algorithm	Future work will focus on improving the detection rate of unknown applications.
Cai et al. [21]	2019	Dynamic feature (method calls and inter-component communication (ICC) intents)	machine learning algorithm	Found that features capturing the app execution structure are much more important than typical security features.
John et al. [22]	2020	Dynamic feature (system calls)	graph convolutional network (GCN)	The first application of a GCN for dynamic Android malware detection.
Taheri et al. [23]	2019	Hybrid feature (permissions, intents, and network-flow)	machine learning algorithm	Plans to generate an Android dataset with more captured features and with a massive sample size.

**Table 2:** The 13 types of Dalvik opcodes.

i	Opcode (hex)	Opcode Keywords	Explanation
1	00	nop	Empty operation instruction: aligns the code and has no actual operation.
2	01-0D	move	Data operation instruction.
3	0E-11	return	Method return instruction: returns the result of the current working function.
4	12-1C	const	Data definition instruction.
5	1D-1E	monitor	Lock instruction: used in multithreaded programs to operate on the same object.
6	1F/20/22	check	Object operation instruction: used to transform, check, and new instances.
7	21/23-26	array	Array operation instruction.
8	27	throw	Exception instruction.
9	28-2C/32-3D	goto/switch/if	Jump instruction: jump from the current address to the specified offset.
10	2D-31	cmpl/cmpg/cmp	Compare instruction: compare the values of two registers.
11	44-6D/F2-F7	iget/iput/sget/sput	Field operation instruction: read or write the fields of the object instance.
12	6E-72/74-78/F0/F8-FB	invoke	Function call instruction: calls the method of other class instances.
13	7B-E2	neg-/not-/int-to/long-to/float-to/double-to/-int/-long/-float/-double	Datatype transfer instruction.

**Table 3:** The mapping relationship obtained from Figure 2 and Figure 3.

The Type of Annotation	The Meaning of the Fields	Fields	Explanation
	Java annotation sign	@requiresPermission	A kind of annotation in Java.
	Number of required permissions	allOf	There are two categories: allOf and anyOf. allOf: all permissions are required. anyOf: only one of the permissions is required.
@RequiresPermission Annotation	Permission name	android.Manifest.permission. MANAGE_DEVICE_ADMINS, android.Manifest.permission. INTERACT_ACROSS_USERS_FULL	MANAGE_DEVICE_ADMINS and INTERACT_ACROSS_USERS_FULL are the required permission names.
	API function name and parameters	setActiveAdmin(@NonNull ComponentName policyReceiver, boolean refreshing, int)	Extract the mapping relationship between the API function name and parameters and permission name based on those fields.
	Permission name	android.Manifest.permission #MASTER_CLEAR	Explanation as above.
@link android.Manifest. permission#XXX Annotation	API function name and parameters	getFactoryResetProtectionPolicy(@Nullable ComponentName admin)	

**Table 4:** Comparison of the subgraph extraction.

Number of Sensitive APIs That Began to Be Extracted	The Average Number of Nodes in the FCG	The Average Number of Nodes in the FCSG	Average Node Reduction Rate	The Total of the Feature Weight in the FCG	The Total of the Feature Weight in the FCSG	Node Feature Reduction Rate
Top 1	12,189	7303	40.1%	1,884,584	1,663,770	11.7%
Top 3		7776	36.2%		1,733,300	8.0%
Top 5		7914	35.0%		1,766,982	6.2%
Top 7		7986	34.5%		1,776,055	5.8%
Top 10		8170	33.0%		1,789,436	5.0%
Top 15		8531	30.0%		1,802,242	4.4%
Top 20		9115	25.2%		1,822,036	3.3%
Top 30		10,094	17.1%		1,866,399	1.0%

**Table 5:** Experimental Results.

GCN Layers	K	Accuracy	Precision	F1 Score	TPR	FPR	AUC
	20	0.9534	0.9696	0.9527	0.9362	0.029	0.9908
2	40	0.9593	0.9644	0.9597	0.9539	0.035	0.9879
	60	0.9719	0.9820	0.9716	0.9614	0.017	0.9962
	20	0.9687	0.9620	0.9688	0.9757	0.038	0.9922
3	40	0.9828	0.9890	0.9827	0.9765	0.011	0.9968
	60	0.9652	0.9625	0.9653	0.9681	0.037	0.9936
	20	0.9631	0.9623	0.9631	0.9640	0.037	0.9930
4	40	0.9476	0.9635	0.9467	0.9304	0.035	0.9867
	60	0.9661	0.9688	0.9660	0.9631	0.031	0.9946

**Table 6:** Comparison of our approach with existing approaches.

Approaches	Accuracy	Precision	F1 Score	TPR	FPR	AUC
Derbin [37]	0.9651	0.9542	0.9431	0.9565	0.043	0.9765
MaMaDroid [39]	0.9681	0.8909	0.8489	0.9371	0.063	0.9599
DAPASA [16]	0.9432	0.9356	0.9422	0.9279	0.072	0.9578
DroidSim [40]	0.9336	0.9124	0.8930	0.8878	0.108	0.9141
AMDroid [41]	0.9749	0.9787	0.9729	0.9739	0.027	0.9748
V et al. proposed [18]	0.9229	0.9242	0.9205	0.9223	N/A	N/A
Ours	0.9828	0.9890	0.9827	0.9765	0.011	0.9968

## Data Availability

Publicly available dataset was analyzed in this study. The research data can be foundon https://www.sec.cs.tu-bs.de/~danarp/drebin (accessed on 5 May 2022), https://www.unb.ca/cic/datasets/maldroid-2020.html (accessed on 17 March 2022) and https://androzoo.uni.lu (accessed on 13 May 2022).

## Abbreviations

The following abbreviations are used in this paper:

GCNs	Graph Convolutional Networks
FCG	Function Call Graph
FCSG	Function Call Subgraph
S-FCSG	Sensitive Function Call Subgraph
AOSP	Android Open Source Project
1-D CNN	1-D Convolutional Neural Network
